# Integration of plasmonic AgPd alloy nanoparticles with single-layer graphitic carbon nitride as Mott-Schottky junction toward photo-promoted H_2_ evolution

**DOI:** 10.1038/s41598-022-17238-4

**Published:** 2022-08-09

**Authors:** Behnam Gholipour, Afsaneh Zonouzi, Mohammadreza Shokouhimehr, Sadegh Rostamnia

**Affiliations:** 1 Department of Chemistry, University of Tehran, P.O. Box 14155-6455, Tehran, Iran; 2 Organic and Nano Group (ONG), Department of Chemistry, Iran University of Science and Technology (IUST), PO Box 16846-13114, Tehran, Iran; 3 Department of Materials Science and Engineering, Research Institute of Advanced Materials, Seoul National University, Seoul, 08826 Republic of Korea

**Keywords:** Chemistry, Catalysis, Energy, Photochemistry

## Abstract

Plasmonic AgPd alloy nanoparticles (AgPd_NPs_) decorated on single-layer carbon nitride (AgPd_NPs_/SLCN) for the designing of the Mott-Schottky junction were constructed with the ultrasonically assisted hydrothermal method and used toward photo evolution H_2_ from formic acid (FA) at near room temperature (30 °C). The Pd atom contains active sites that are synergistically boosted by the localized surface plasmon resonance (LSPR) effect of Ag atoms, leading to considerably enhanced photocatalytic properties. The photoactive AgPd_NPs_/SLCN obtained supreme catalytic activity to produce 50 mL of gas (H_2_ + CO_2_) with the initial turnover frequency of 224 h^-1^ under light irradiation. The catalyst showed stable catalytic performance during successive cycles.

## Introduction

Access to alternative and clean energy sources has become one of the most important issues due to the increasing demand for energy and the reduction of the use of conventional fossil fuels^1–3^. Due to the exhaustion of fossil fuels and ecological issues, hydrogen-based fuel is one of the most hopeful clean and sustainable energy sources for current society^4–8^. Currently, the industrial method for the production of hydrogen is mainly done through vapor reforming and coal gasification, which is based on confined fossil sources such as natural gas, coal, and oil. Based on mid to long-term plans, there is a growing requirement for substitute feedstocks for the production of H_2_ in a more sustainable way^9,10^. In recent years, various chemical hydrogen storing materials, such as methanol, formic acid, formaldehyde and boron ammonia have been extensively studied^11–22^. Formic acid (FA) as a hopeful material in H_2_ production/storage has engrossed much consideration owing to its great gravimetric/volumetric H_2_ capacity, easiness of use, non-toxicity, ambient temperature stability and plentiful supply from the transformation of biomass and carbon dioxide^23–28^.

For the use of FA as a liquid organic H_2_ carrier (LOHC), the expansion of competent catalysts, especially heterogeneous catalysts for FA decomposition, is a challenging issue^29,30^. On the other hand, over the past decades, the field of heterogeneous photocatalysis has extended swiftly and has faced various developments, particularly in regard to energy and the environment. Accordingly, in recent years, photocatalytic degradation of FA to CO_2_ and H_2_ has been broadly studied in the literature^31–35^. Recent reports have shown that the design and manufacture of conjugated semiconductor polymers due to their advantages such as constancy in aqueous-medium, visible light absorbency, intramolecular charge transition, the low expense is one of the effective strategies^36–38^. Supported semiconductors with visible light absorption can be used to modify the photocatalytic operation of heterogeneous metal based catalysts for photo- decomposition formic acid^31,32,35,39,40^. This is owing to the electronic interaction and electron handover between the metal and the semiconductor due to the Mott–Schottky effect on the surface of the metal based semiconductor interfaces^41^. The integration of plasmonic based alloy (AgPd and AuPd) and semiconductors to make Mott-Schottky photocatalysts is an effective manner to improve photocatalytic operation by accelerating the charge kinetics of photocatalytic reactions^32,35,42^.

Carbon nitride (g-C_3_N_4_) has been widely used as one of the most important photocatalyst in the production of hydrogen by visible light^43–46^. This is due to the π-conjugated graphitic carbon nitride structure that provides a specific electronic property for charge transfer^47–50^. On the other hand, as an active semiconductor in visible light, it has a relatively narrow bandgap of 2.7 eV and favorable conduction band (C_b_) and valence band (V_b_), which is appropriate for UV–vis light absorption for hydrogen evolution^47,51–59^. The most important point is that the performance of most noble metals is betwixt the C_b_ and the V_b_ of graphitic carbon nitride, which leads to the high reinforcement of the handover of photogenerated electrons from graphitic carbon nitride to metal nanoparticles owing to the Mott-Schottky effect ^32,35^. In 2017, Wu et al. reported the monodisperse of AgPd alloy on graphite carbon nitride semiconductor (AgPd/CN) for photocatalytic evolution of hydrogen from formic acid^31^. Yu et al. offered the Mott-Schottky heterojunction based on AgPd NWs and g-C_3_N_4_ for photocatalytic dehydrogenation of FA in the presence of visible light (λ > 400 nm)^35^. Recently, Cheng et al. reported graphite carbon nitride nanosheet containing AgPd bimetallic nanoparticles with Ag plasmonic effect as an effective Mott-Schottky photocatalyst to catalyzing the evolution of H_2_ from the formic acid under visible light^42^. Compared to bulk g-C_3_N_4_, the single layer carbon nitride is an emerging photocatalyst with a layered structure due to its unique properties such as photogenerated charge carrier lifetime, specific surface area, shorter bulk diffusion length, and high carrier density reduces the possibility of recombination of charge carriers and affords more surface-active sites for metal stabilization^60–63^. Inspired by the aforesaid considerations and in line with our recent efforts in the field of clean energy and hydrogen production^28,64–69^, here we offer the synthesis of bimetallic AgPd_NPs_ decorated on single layer carbon nitride (SLCN) as an active plasmonic photocatalyst for the evolution of H_2_ from FA.

## Experimental

### Materials and methods

Melamine (Aldrich, 99%), AgNO_3_ (Aldrich, 99.99%), K_2_PdCl_4_ (Aldrich, 99.9%), Formic acid (Merck, 98–100%), KCl (Merck, 99.99%), NaBH_4_ (Merck, 99%) were used without additional refinement. Ultrapure water (18.5 MΩ Milli Q) was utilized for all experimentations.

### Characterization

FT-IR spectrums were recorded by a Shimadzu IR-460 spectrometer. Powder X-ray diffraction (PXRD) patterns were performed for samples using the D_8_ ADVANCE X-ray diffractometer diffraction system with Cu Kα radiation (λ = 1.5406 Å). UV–vis absorption and diffuse reflectance spectra were recorded using SHIMADZU, UV -2450. The surface morphology of samples was determined by the Hitachi S4700 FE-SEM. TEM images were captured with a Zeiss EM 900 electron microscope. The generated gas molecules (H_2_ + CO_2_) were monitored by gas chromatography (GC) armed with a TCD detector and the results were contrasted with the automatic CO_2_ gas measuring apparatus.

### Synthesis of g-C_3_N_4_

Synthesis of g-C_3_N_4_ using thermal polymerization of melamine was carried out in an alumina crucible with a cover, from ambient temperature up to 550 °C at a heating rate of 2.3 °C per minute under air conditions in a muffle furnace. In the following, by keeping the temperature constant at 550 °C for 4 h the yellow g-C_3_N_4_ was obtained^70^.

### Synthesis of SLCN

Synthesis of g-C_3_N_4_ was executed using a complementary two-step manner (ultrasonic-hydrothermal method). For this purpose, 0.1 g of as-made bulk g-C_3_N_4_ was first exposed to ultrasonic waves for 2 h. In the following, after resting the obtained solution for 5 min, the upper part of the solution was separated and rendered into a Teflon-lined stainless autoclave (100 ml) and then heated at 120 °C for 10 h. Eventually, after the desired time had elapsed and the autoclave temperature reached ambient temperature, the almost yellowish white mixture was isolated by centrifugation (5000 rpm) and dried up at 50 °C for 12 h.

### Synthesis of AgPd_NPs_/SLCN

For the synthesis of AgPd_NPs_/SLCN, 0.1 g SLCN was dispersed in 40 mL distilled water and then sonicated at 25 °C for 30 min. Subsequently aqueous AgNO_3_ and K_2_PdCl_4_ (mole ratio = 1:0, 2:1, 1:1, 1:2, 0:1) was extra, and then magnetically stirred for overnight at ambient temperature. Afterward, 2.5 mL of aqueous NaBH_4_ (0.15 M) was added drop by drop into the mixture and stirred for another 2 h. After 2 h, the samples were gathered by centrifugation (5000 rpm) and rinsed with distilled water several times. The samples were dried in an oven at 50 °C for 15 h.

### Photocatalytic dehydrogenation of FA

The photocatalytic H_2_ evolution assessment was performed in a closed 50 mL reactor by a 24 W LED-SMD lamp. For this purpose, in 10 ml of deionized water, 20 mg of AgPd_NPs_/SLCN catalyst was dispersed by sonication and the reactor oxygen was purged by N_2_ before the reaction. Then 0.38 ml of 98% FA was injected into the catalyst solution at room temperature. The gas molecules produced were analyzed by a gas chromatograph. The amount of gas (H_2_ + CO_2_) evolution during the photocatalytic reaction was evaluated by a gas burette system. The TOFs are calculated within the initial 10 min conforming to the subsequent Eq. ^32^:

$${{TOF = n}}_{{{gas \,produced}}} {{ /(nAgPd \times h)}}$$

## Results and discussion

**Figure 1:**
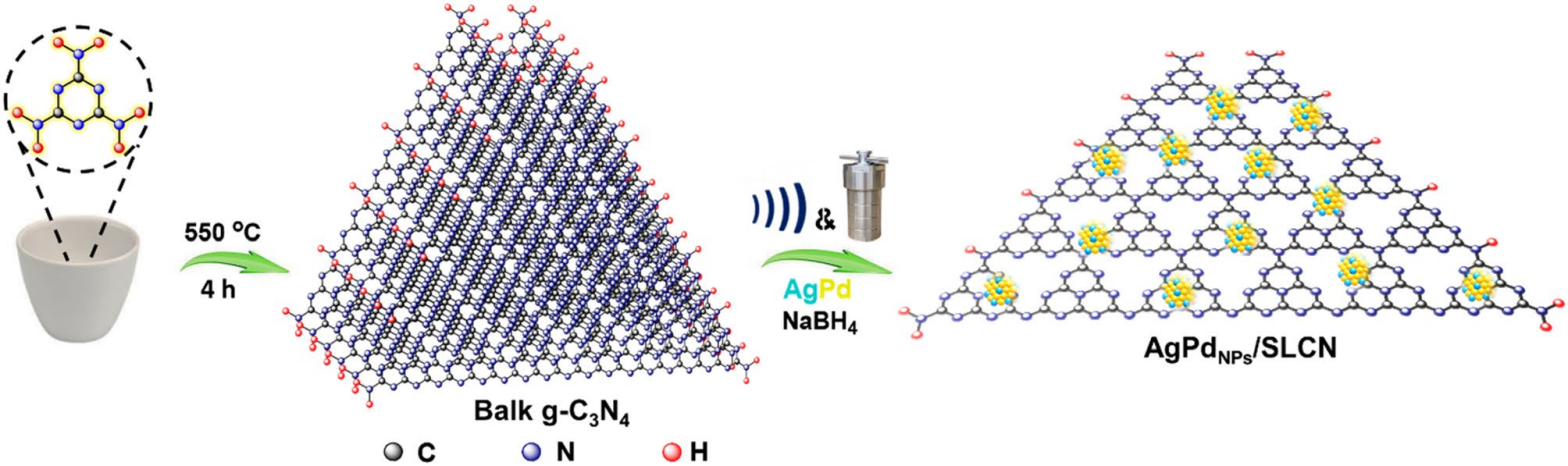
Schematic illustration for the synthesis of AgPd_NPs_/SLCN.

As shown in Fig. 1, we constructed the Mott-Schottky junction based ultra-thin carbon nitride single layers decorated with AgPd alloy nanoparticles with a simple strategy through a strong interaction between AgPd_NPs_ and SLCN toward enhancement photocatalytic dehydrogenation of FA.

**Figure 2:**
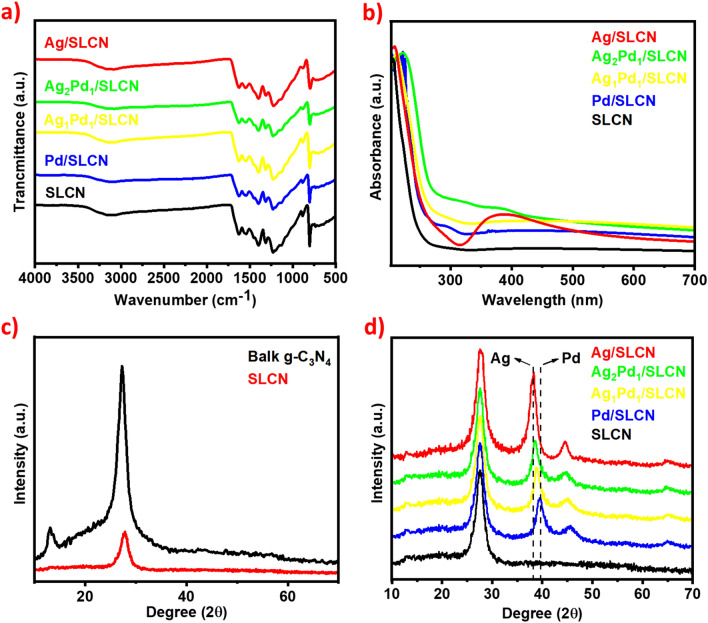
(**a**) FT-IR spectrum, (**b**) UV–vis spectra of SLCN, Ag/SLCN, Ag_2_Pd_1_/SLCN, Ag_1_Pd_1_/SLCN and Pd/SLCN, (**c**) XRD patterns of the bulk g-C_3_N_4_ and SLCN, (**d**) XRD patterns of SLCN, Ag/SLCN, Ag_2_Pd_1_/SLCN, Ag_1_Pd_1_/SLCN and Pd/SLCN.

The chemical structure of SLCN as well as SLCN decorated with AgPd nanoparticles was analyzed by FT-IR spectrum (Fig. 2a). The band appeared at 809 cm^-1^ is ascribed to the specific breathing mode of the triazine ring. Multiple peaks in the proximity to each other and in the range of 1200–1700 cm^-1^ are related to the stretching modes of aromatic C-N heterocycles. Wide absorption in the area of 3000–3500 cm^-1^ is attributed to the N–H stretching mode^71^. UV–vis absorption spectra of AgPd_NPs_/SLCN samples were inspected (Fig. 2b). For Ag nanoparticles, the LSPR with high intensity in a region of 390–410 nm is observed. In the case of AgPd alloy, the intensity of the peak’s changes with a change in the Ag:Pd ratio, so that for a ratio of 1:1 compared to 2:1, the effect of LSPR shows a greater reduction, which can be due to charge transfer to the Ag_NPs_ surface^37,72–74^. XRD planes of the bulk g-C_3_N_4_, SLCN and AgPd alloy nanoparticles supported on SLCN with different Ag:Pd ratios were revealed in Fig. 2c,d. For bulk g-C_3_N_4_, a slight peak at 13.1° is observed, which is assigned to the (100) plan, attribute to the in-plane structural packaging motif of tri-s-triazine units. The strong diffraction at 27.5° also belongs to the (002) plan, which is related to the accumulation between the inter-layer stacking of conjugated aromatic rings. The peak with low intensity at 27.5° is related to the SLCN, which indicates the interlayer structure of bulk CN was annihilated after exfoliation^71^. For AgPd alloy nanoparticles with different ratios, the peak appears at a 39°, which shifted to elder angles with increasing Pd:Ag ratio, signifying that the AuPd alloy is formed on SLCN^31^. The nitrogen adsorption–desorption isotherms for SLCN show type III isotherms (Fig. S1). The specific surface area of SLCN is 88.05 m^2^/g, which is 14.5 times larger than bulk g-C_3_N_4_ (6.06 m^2^/g). The pore size distribution of BJH shows an average pore diameter of 1.64 and 14.37 nm for SLCN and 12.24 nm for bulk CN (Fig. S2).The high specific surface area and large total pore volume indicate that SLCN has a nanoporous structure, leading to increased photocatalytic performance through favorable mass transfer.

**Figure 3:**
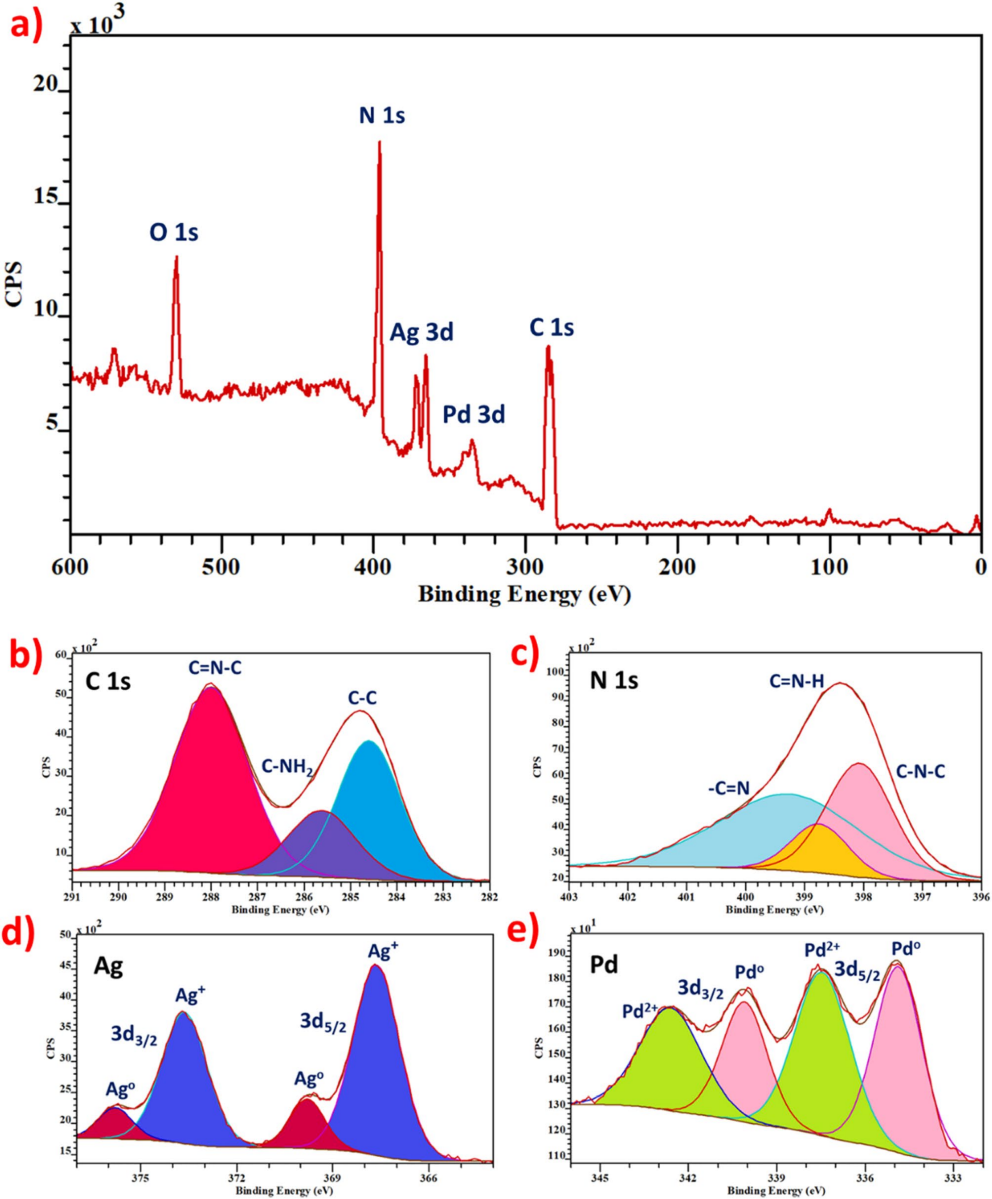
(**a**) general XPS of Ag_2_Pd_1_/SLCN, (**b**) C1s regions of Ag_2_Pd_1_/SLCN, (**c**) N1s regions of Ag_2_Pd_1_/SLCN (**d**), Ag 3d regions of Ag_2_Pd_1_/SLCN and (**e**) Pd 3d regions of Ag_2_Pd_1_/SLCN.

Figure 3a demonstrates the general spectrum of Ag_2_Pd_1_/SLCN in which the characteristic peaks C 1 s, N 1 s, Ag 3d and Pd 3d are clearly visible. In the high-resolution XPS spectrum for the C element, the binding energy at 284.7 eV is attributed to the C–C bond and at 288 eV to C = N–C at SLCN (Fig. 3b)^75–77^. Figure 3c also shows the XPS spectrum of element N in Ag_2_Pd_1_/SLCN, which corresponds to the energy bands C–N–C and C = N–H and –C = N^75–77^. Photoelectron spectroscopy (XPS) analysis was executed to further explore the effect of SLCN on the electronics of Ag and Pd structures in Ag_2_Pd_1_/SLCN. Examination of the XPS spectra of Ag 3d and Pd 3d shows that the binding energies for Pd 3d_3/2_ in Ag_2_Pd_1_/SLCN is shifted to lower values due to the handover of electrons from SLCN and Ag to Pd, while the binding energies for Ag 3d_3/2_ in Ag_2_Pd_1_/SLCN shift to higher values (Fig. 3d,e)^31,35,78^. In fact, this indicates the redistribution of charge from higher Fermi level (Ag) to lower Fermi level (Pd)^31^. According to XPS analysis, the atomic percentages of elements in Ag_2_Pd_1_/SLCN for C, N, O, Ag and Pd are 32.82%, 60.04%, 6.52%, 0.42% and 0.2%, respectively.

**Figure 4:**
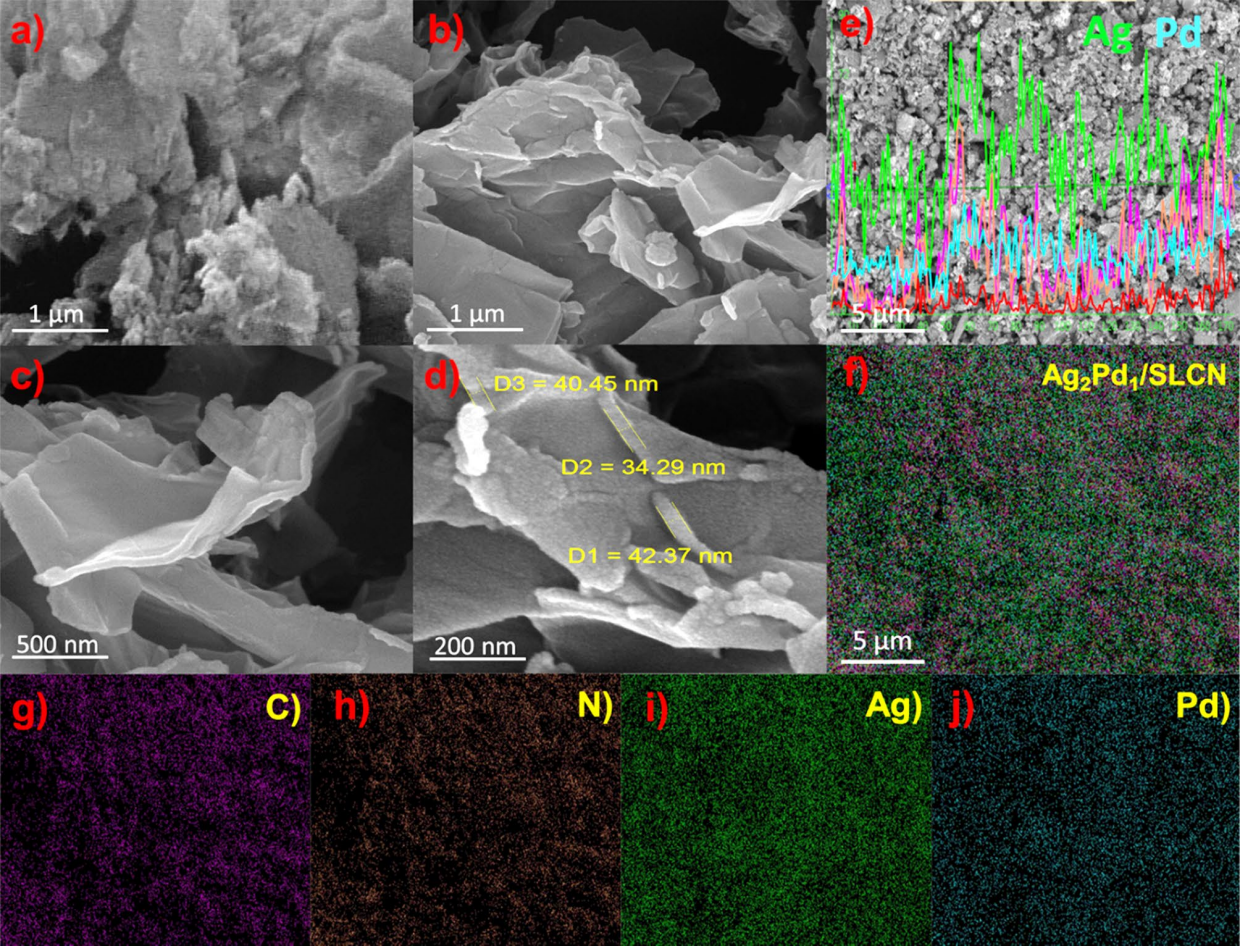
(**a**) SEM images of bulk g-C_3_N_4_, (**b**, **c** and **d**) SEM images of SLCN, (**e**) EDS-SEM line scan profiles of Ag_2_Pd_1_/SLCN, (**f**) general map of Ag_2_Pd_1_/SLCN and (**g**–**j**) elemental mappings of SEM–EDX mappings of Ag_2_Pd_1_/SLCN.

Figure 4a–d exhibitions the SEM image of the bulk g-C_3_N_4_ and SL-CN after ultrasonic exfoliation and then hydrothermal procedure. The presence of many sheets with laminar morphology proves SLCN synthesis. The SEM–EDS image and elemental mapping images approve the attendance of all composing elements (C, N, Ag, Pd) in the Ag_2_Pd_1_/SLCN structure (Fig. 4f–j). Figure 4e displays the EDS-SEM line scan profiles of Ag_2_Pd_1_/SLCN containing C, N, Ag and Pd atoms, which clearly confirm the approximately homogeneous distribution of atoms.

**Figure 5:**
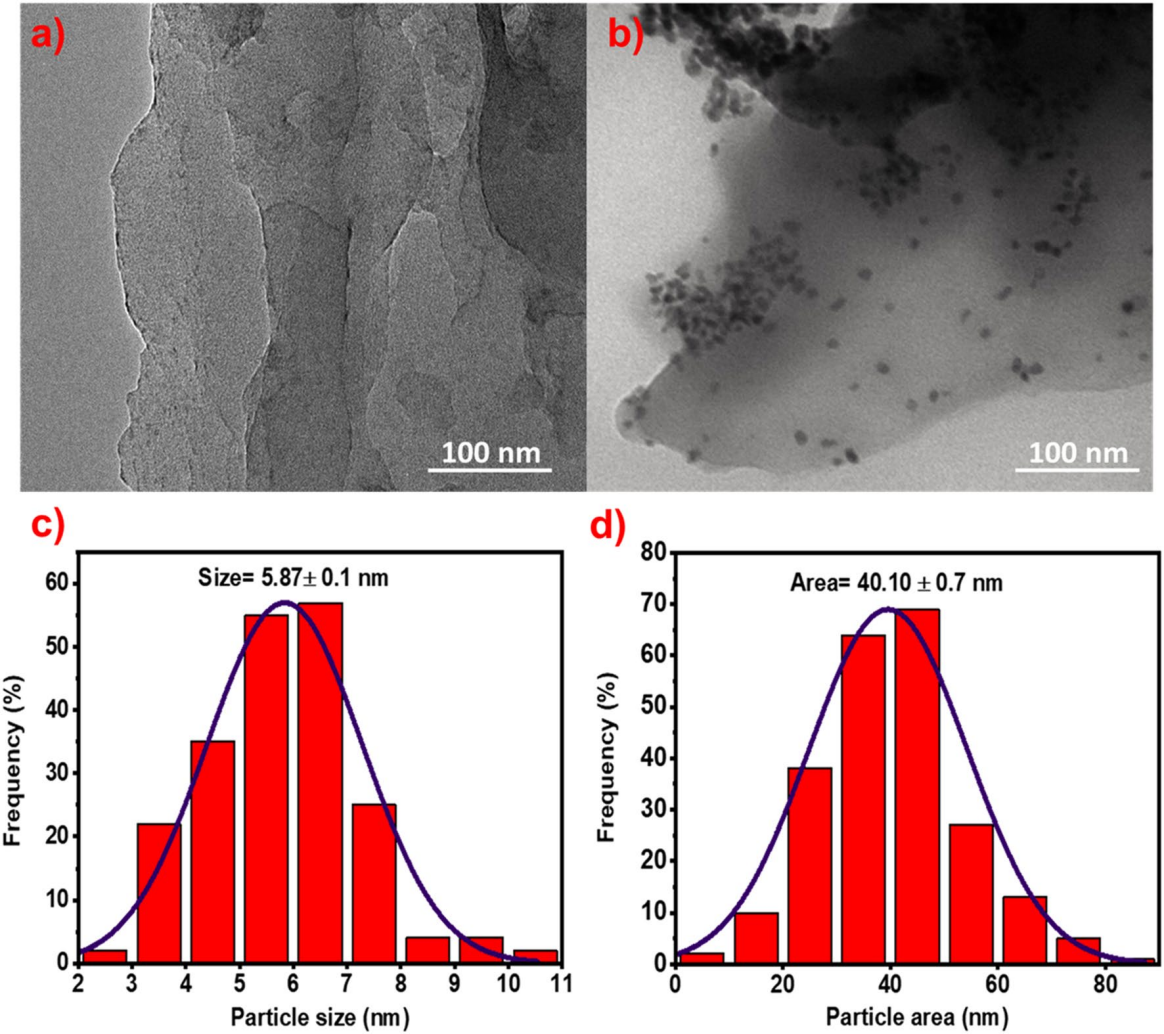
(**a**) TEM image of SLCN, (**b**) TEM image of Ag_1_Pd_1_/SLCN, (**c**) the average diameter histogram of AgPd_NPs_ for Ag_1_Pd_1_/SLCN and (**d**) the average particle area histogram of AgPd_NPs_ for Ag_1_Pd_1_/SLCN.

The TEM image and related particle size histogram for SLCN and AgPd_NPs_/SLCN (Ag: Pd in a ratio of 1: 1) is revealed in Fig. 5a–d. As is clear, a single-layer structure is beheld for the SLCN, which is also concord with the SEM image. The TEM image also shows 6 nm spherical AgPd_NPs_ that are well embedded on the surface of SLCN.

The photoluminescence (PL) spectra (excitation under 325 nm) for SLCN and Ag_1_Pd_1_/SLCN are provided in Fig. S2. The PL intensity of Ag_1_Pd_1_/SLCN, is lower than that of SLCN, indicating that compared to SLCN, photo-generated electron transfer in Ag_1_Pd_1_/SLCN accelerates faster and diminishes the electron recombination rate of electrons and holes. Compared to SLCN and Ag_1_Pd_1_/SLCN, the peak intensity is higher for g-C_3_N_4_, which indicates that the recombination rate of electrons and holes is higher (Fig. S3). UV-DRS measurement was also performed to evaluate the optical absorption properties of SLCN and Ag_1_Pd_1_/SLCN (Fig. S4). For SLCN, low absorption between 450 and 700 nm is observed, indicating the poor performance of SLCN in visible light. After modification of SLCN with AgPd nanoparticles, the absorption of visible light increased slightly, indicating the acceptable performance of Ag_1_Pd_1_/SLCN in visible light. Also, the presence of poor absorption in the range of 450–600 nm could be related to the localized surface plasma resonance (LSPR) effect of silver nanoparticles on the surface of SLCN.

The optical bandgap for the photocatalysts was obtained using the Tauc’s equation^79^:

$${{\alpha h\upsilon = A}}\left( {{{h\upsilon - E}}_{{{g}}} } \right)^{{{n}}}$$

α: is the absorption coefficient. E_g_: band gap. h: Planck’s constant. υ: the frequency of light. n: the electron transition process constant (the value of n is considered to be as 1/2). A: constant (in the ideal case A = 1).

**Figure 6:**
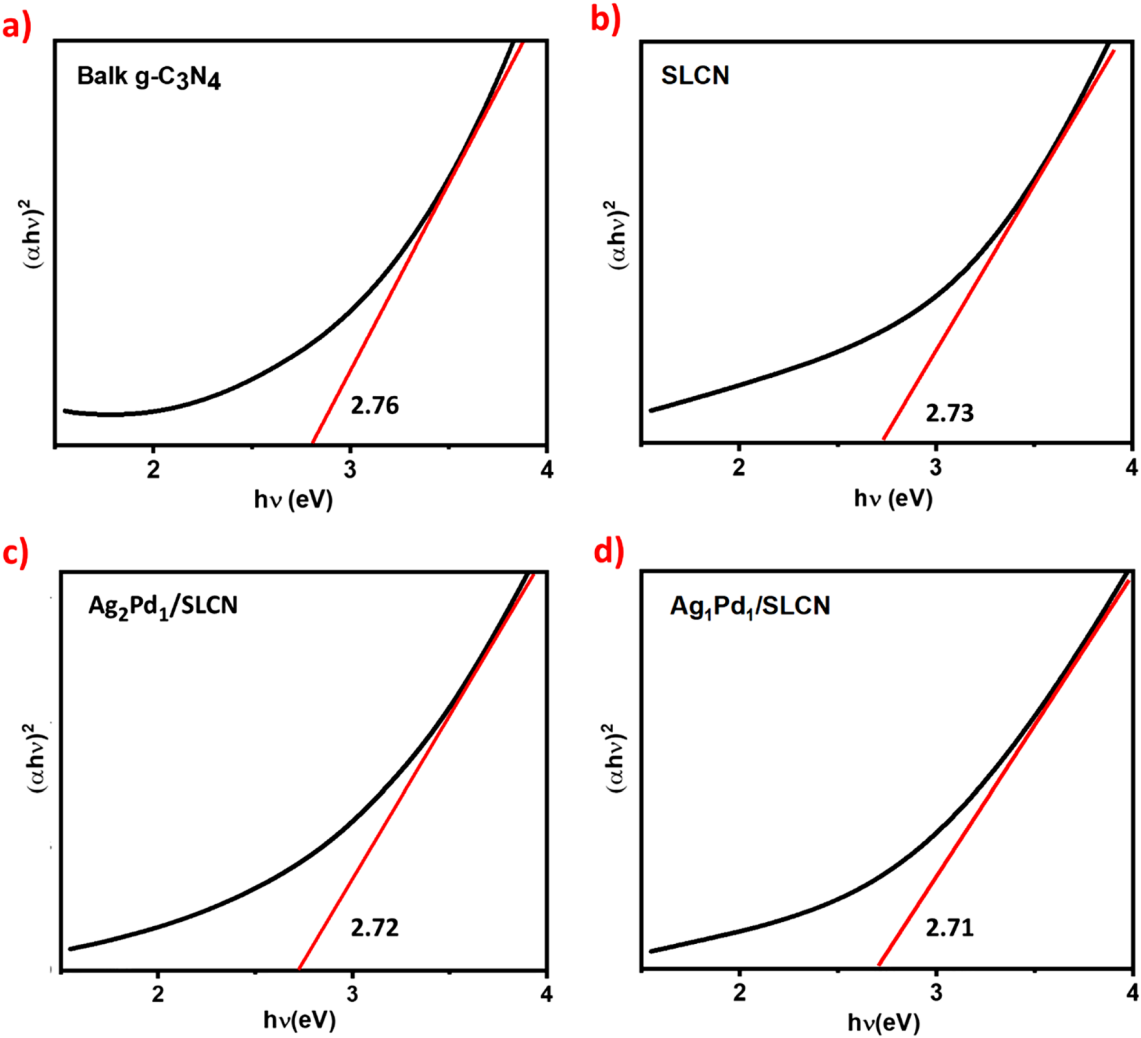
(**a**–**d**) plots of (αhυ)^2^ versus hυ for bulk g-C_3_N_4_, SLCN, Ag_2_Pd_1_/SLCN and Ag_1_Pd_1_/SLCN.

For each of the desired photocatalysts, the optical energy bandgap was appointed by extrapolation of the lined area of the plot of (αhυ)^2^ versus hυ (Fig. 6a–d).

**Figure 7:**
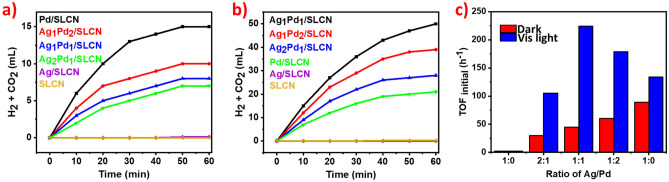
(**a**) Time-dependent gas evolution plots from FA in the dark at ~ 30 °C, (**b**) in the light irradiation at ~ 30 °C and (**c**) the TOFs ratio of various catalysts in the dark and light irradiation.

Catalytic H_2_ evolution for 10 mL of 1.0 M aqueous FA was tested for all synthesized samples in the utilization of 0.2 g of the catalyst under dark conditions at ambient temperature. No gas was produced for SLCN or Ag/SLCN. In comparison, when Pd/SLCN, AgPd/SLCN were used, gas release was observed within 60 min, which the highest production value (15 mL) was obtained for Pd/SLCN (Fig. 7a). Examination of the above conditions under visible light irradiation showed a comparable increased activity compared to the dark conditions for all Pd-comprising photocatalysts. Among the bimetallic photocatalysts for the 1: 1 ratio of Ag/Pd, the amount of gas produced was 50 mL, which shows the highest evolution rate compared to the 1: 3 and 3: 1 ratio (39 and 28 mL, respectively) (Fig. 7b). Specifically, the activity of bimetallic AgPd_NPs_ photocatalysts is superior to that of Pd_NPs_ under the same situations. This can be credited to the higher activity of AgPd alloys which occurs as a result of stout-interplay and charge redistribution betwixt Ag and Pd and thus accelerate the photocatalytic dehydrogenation of FA. Figure 7c illustrations the TOFs value versus the ratio of Ag:Pd for the SLCN-supported photocatalysts in both dark and visible light conditions. Specifically, Ag_1_Pd_1_/SLCN disclosed the upmost activity with a TOF value of 224 h^-1^ under visible light irradiation. The highest TOF value in the dark condition was also obtained for Pd/SLCN, which was equal to 53 h^-1^. The results specify that alloying Ag with Pd leads to a synergistic effect and thus increases the activity of the desired photocatalyst. Accordingly, Ag_1_Pd_1_/SLCN was the best photocatalyst among the catalysts we studied for the evolution of hydrogen from the FA.

**Figure 8:**
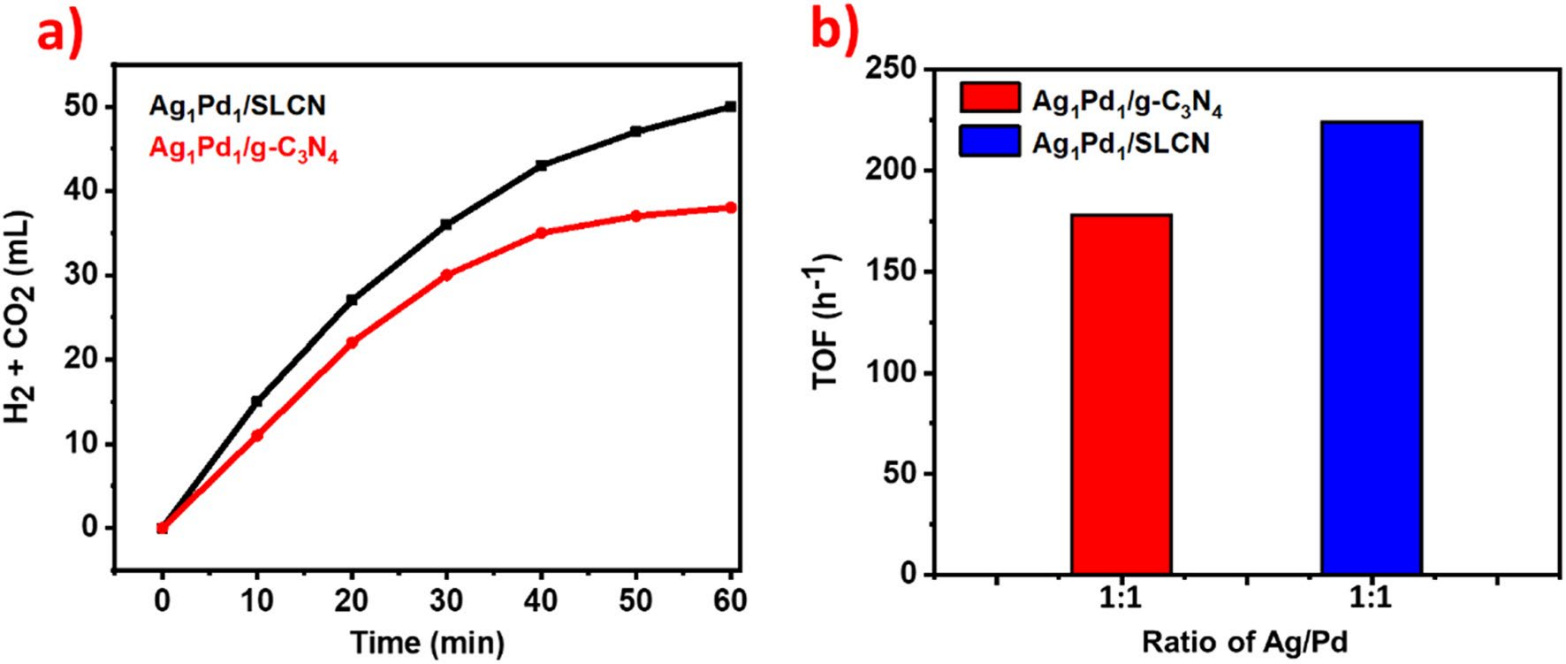
(**a**) Time-dependent gas evolution plots from FA in the light irradiation at ~ 30 °C for Ag_1_Pd_1_/SLCN versus Ag_1_Pd_1_/g-C_3_N_4_ (**b**) the TOFs ratio of Ag_1_Pd_1_/SLCN versus Ag_1_Pd_1_/g-C_3_N_4_ in the light irradiation.

Support of AgPd alloy on bulk carbon nitride (Ag_1_Pd_1_/g-C_3_N_4_) was evaluated to investigate the Mott-Schottky effect on photocatalytic performance compared to Ag_1_Pd_1_/SLCN. As shown in Fig. 8a, less gas evolution (38 mL) was observed for Ag_1_Pd_1_/g-C_3_N_4_ under light irradiation. It is clear that the catalytic performance of Ag_1_Pd_1_/SLCN is higher than that of Ag_1_Pd_1_/g-C_3_N_4_, mainly owing to the large specific surface area of the SLCN, which effectively shortens the electron transfer path between the support and the AgPd. For Ag_1_Pd_1_/g-C_3_N_4_ the photocatalytic activity with a TOF value of 178 h^-1^ was obtained as shown in Fig. 8b.

**Figure 9:**
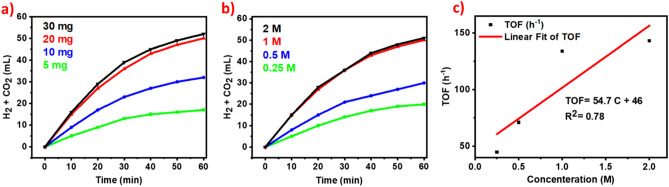
(**a**) The volume of gas evolution in the use of different amount of Ag_1_Pd_1_/SLCN for the photo-decomposition of FA, (**b**) the volume of gas evolution in the use of different concentrations for the photocatalytic decomposition of FA and (**c**) Initial TOF versus concentrations for Ag_1_Pd_1_/SLCN under light irradiation.

Based on the above findings, we focused our studies in line with the influence of different amounts of Ag_1_Pd_1_/SLCN catalysts for the photocatalytic evolution of hydrogen. Accordingly, we tested values of less and more than 20 mg of Ag_1_Pd_1_/SLCN catalyst for achieving a better result. Using the values of 5 and 10 mg of catalyst showed less gas evolution. For 30 mg of the catalyst, a similar result was obtained with the amount of 20 mg catalyst (Fig. 9a). In the following, by achieving the optimal amount of catalyst, we also studied the effect of different concentrations of FA. It is necessary to mention this point, we performed all of our studies at a concentration of 1 M, 10 mL of formic acid in accordance with valid reports in the literature. However, due to our interest in completing our research results, we also evaluated different concentrations of formic acid. At low concentrations, for example, 0.25 and 0.5 M, the amount of gas production was lower (20 and 30 ml, respectively), while at a concentration of 2 M, the amount of gas produced was 51 mL. This specifies that for high concentrations the evolution of hydrogen with increased intensity is not observed (Fig. 9b). The rate of photocatalytic dehydrogenation of FA augmented linearly with increasing concentration (Fig. 9c).

**Figure 10:**
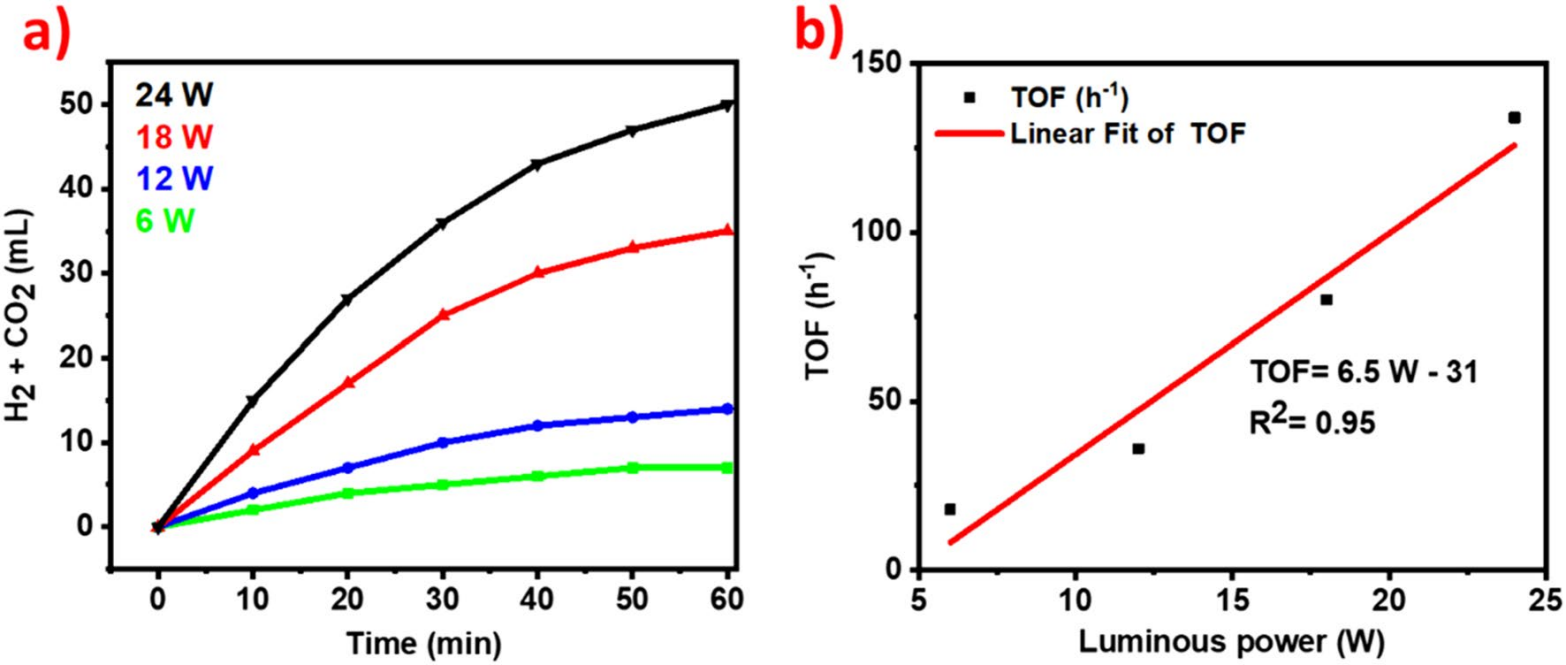
(**a**) The volume of gas evolution in the use of different light intensities for the photocatalytic decomposition of FA and (**b**) Initial TOF versus luminous power for Ag_1_Pd_1_/SLCN under visible light irradiation.

In the next step, the effect of light intensity on photo-decomposition of FA was explored using Ag_1_Pd_1_/SLCN and it was observed that the rate of dehydrogenation of FA enlarged linearly with increment light intensity (Fig. 10a,b).

**Figure 11:**
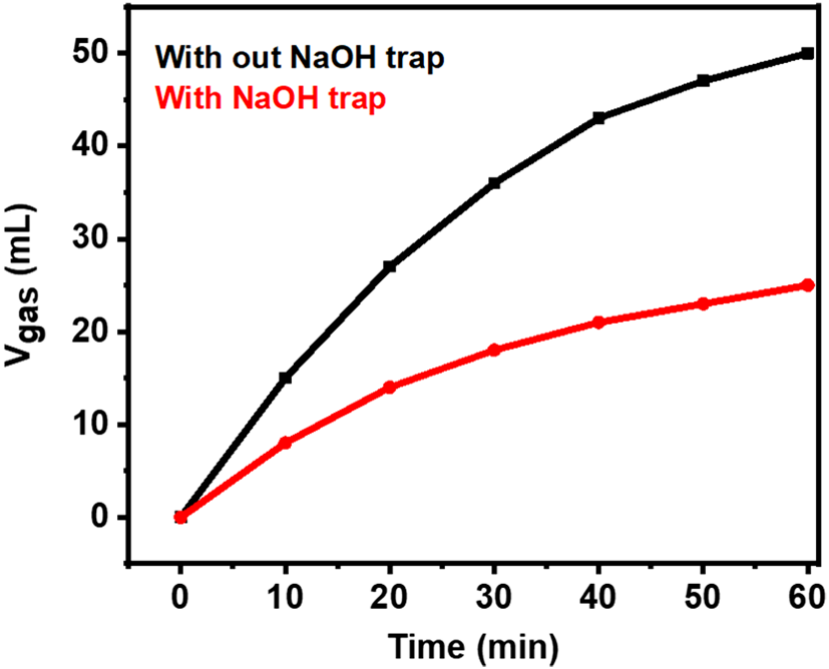
The comparison of the volume of gas produced by using Ag_1_Pd_1_/SLCN for dehydrogenation of aqueous FA solution with and without NaOH trap under visible light irradiation.

To ascertain the molar ratio of CO_2_:H_2_, a NaOH trap (10 M NaOH based on a gas burette system) was utilized to absorb CO_2_^35,80^. In this experiment, the gas mixture produced was passed through a NaOH trap. The volume of gas produced in the use of the NaOH trap was reduced by half compared to the state without traps, which indicates that the volume of gas produced is 1: 1 (Fig. 11).

**Figure 12:**
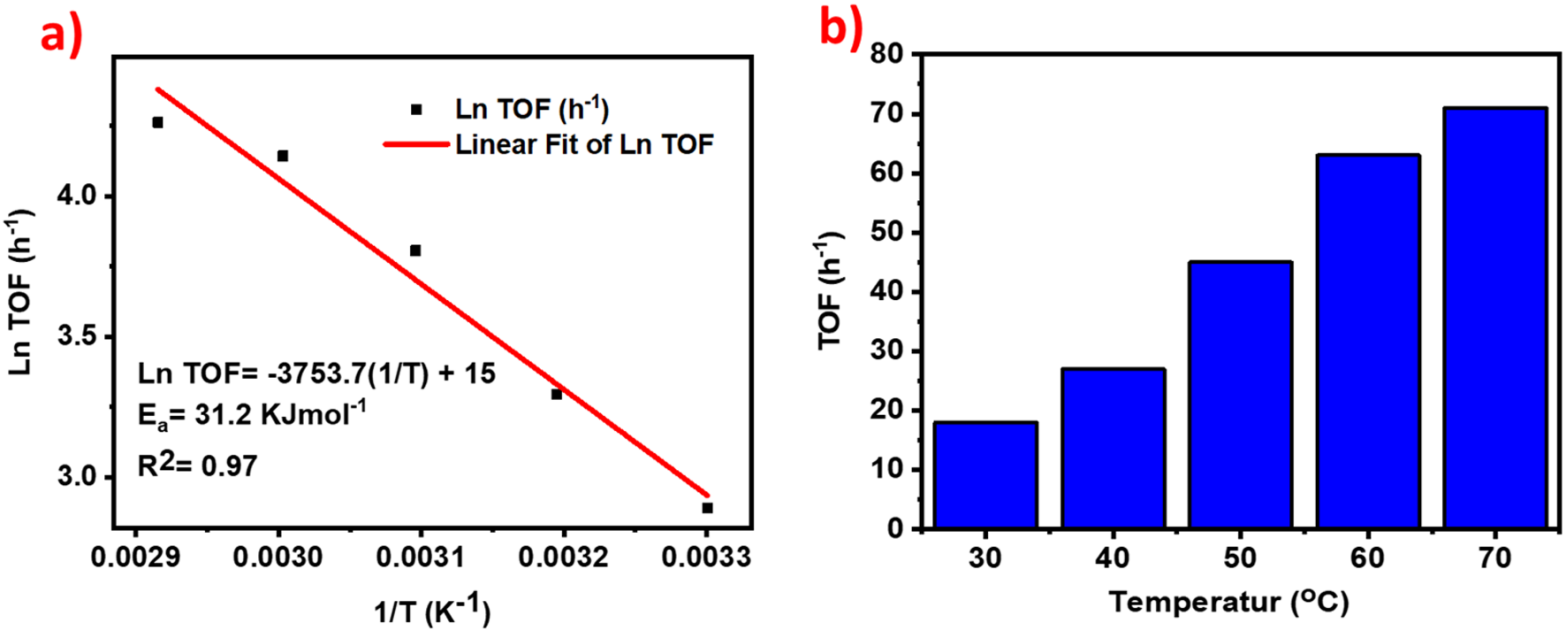
(**a**) Ln TOF versus 1/T plot in course of FA dehydrogenation by Ag_1_Pd_1_/SLCN at diverse temperatures, (**b**) Initial TOF versus temperatures plot for Ag_1_Pd_1_/SLCN.

The activation energy (Ea =  + 31.2 KJmol^−1^) of reaction for Ag_1_Pd_1_/SLCN catalyst was calculated from the (Ln TOF = −Ea/R + (1/T) + Ln A, R = 8.314 J/mol. K) relationship (Fig. 12b). The value of R-square 0.97 was attained, which indicates that the points are the lined trend line.

**Figure 13:**
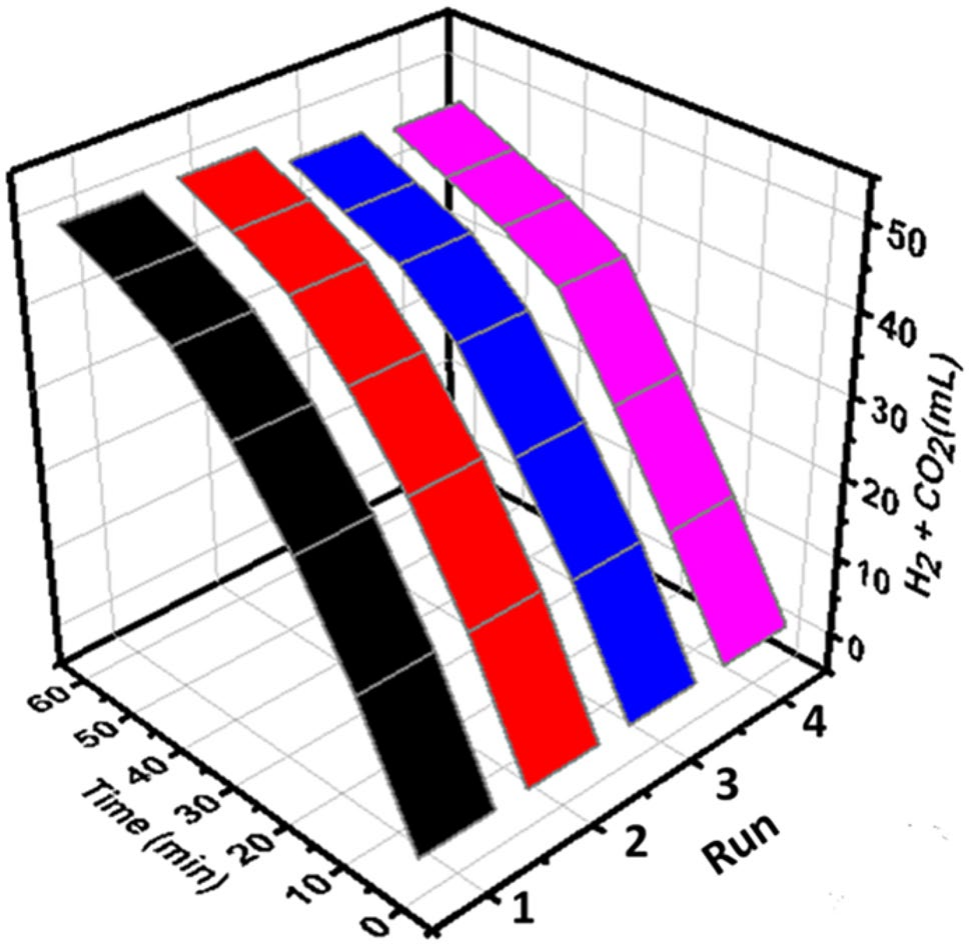
The recycling capability of Ag_1_Pd_1_/SLCN below visible light irradiation.

Ag_1_Pd_1_/SLCN activity after four times exposure to visible light exposed that this catalyst is entirely stable and recyclable below visible light irradiation (Fig. 13).

Table 1 summarizes the results of our work compared to AgPd/C_3_N_4_-based photocatalysts previously reported in the literature. As can be seen, the Ag_1_Pd_1_/SLCN photocatalyst with active sites, showed acceptable catalytic activity relative to the reported works.

**Table 1:** Comparison of different AgPd/C_3_N_4_-based photocatalysts for H_2_ evolution.

Entry	Catalyst	Lamp	T (^o^C)	TOF (h^-1^)	V_gas_ (mL)	Ref
1	AgPd/CN	300 W Xenon	30	254	25	^31^
2	PdAg@g-C_3_N_4_	300 W Xenon	25	420	330	^35^
3	AgPd/2D CNNs	300 W Xenon	50	2936.8	135	^42^
4	AgPd/SLCN	24 W LED-SMD	30	224	50	This work

## Conclusions

In summary, a series of AgPd alloy nanoparticles decorated on an SLCN semiconductor surface was designed to optimize plasmonic Mutt-Schottky catalysts toward the competent evolution of photocatalytic hydrogen from FA. Exhaustive studies revealed that the being of coordinated unsaturated N atoms on the SLCN surface is indispensable for the concurrent stabilization of Ag and Pd as uniform alloy nanoparticles. On the other hand, the desirable charge handover from SLCN and Ag to Pd leads to the enrichment of Pd with electrons and thus affords more catalytic activity and stability for the H_2_ evolution below visible light. This study presents a new path for designing plasmonic matte-Schottky heterogeneous catalysts with synergistic effects and high efficiency for photocatalytic hydrogen evolution by using formic acid.

## Supplementary Information

Supplementary Information.

## Data Availability

All data generated or analysed during this study are included in this published article (and its Supplementary Information files).
