# Supplementary material for: Integration of plasmonic AgPd alloy nanoparticles with single-layer graphitic carbon nitride as Mott-Schottky junction toward photo-promoted H2 evolution
Source: Sci Rep. 2022 Aug 9;12:13583. doi: 10.1038/s41598-022-17238-4 (PMC9363438; doi:10.1038/s41598-022-17238-4)
Supplement: Supplementary file 1 — Supplementary Information. [file 41598_2022_17238_MOESM1_ESM.docx]

**Integration of plasmonic AgPd alloy nanoparticles with single-layer graphitic carbon nitride as Mott-Schottky junction toward Photo-promoted H_2_ evolution**

Behnam Gholipour, ^a^ Afsaneh Zonouzi, ^a,^* Mohammadreza Shokouhimehr, ^c^ Sadegh Rostamnia ^b,^*

*^a^ Department of Chemistry, University of Tehran, P.O. Box 14155-6455, Tehran, Iran*

*Email: zonouziafsaneh@ut.ac.ir*

*^b^ Organic and Nano Group (ONG), Department of Chemistry, Iran University of Science and Technology (IUST), PO Box 16846-13114, Tehran, Iran Email: rostamnia@iust.ac.ir; srostamnia@gmail.com*

*^c^ Department of Materials Science and Engineering, Research Institute of Advanced Materials, Seoul National University, Seoul 08826, Republic of Korea*


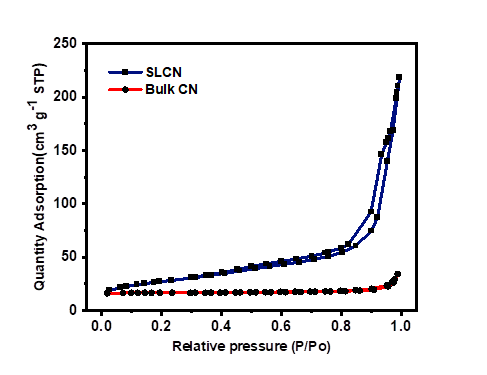


**Fig S1.** The nitrogen adsorption-desorption isotherms of bulk CN and SLCN^S1,2^.


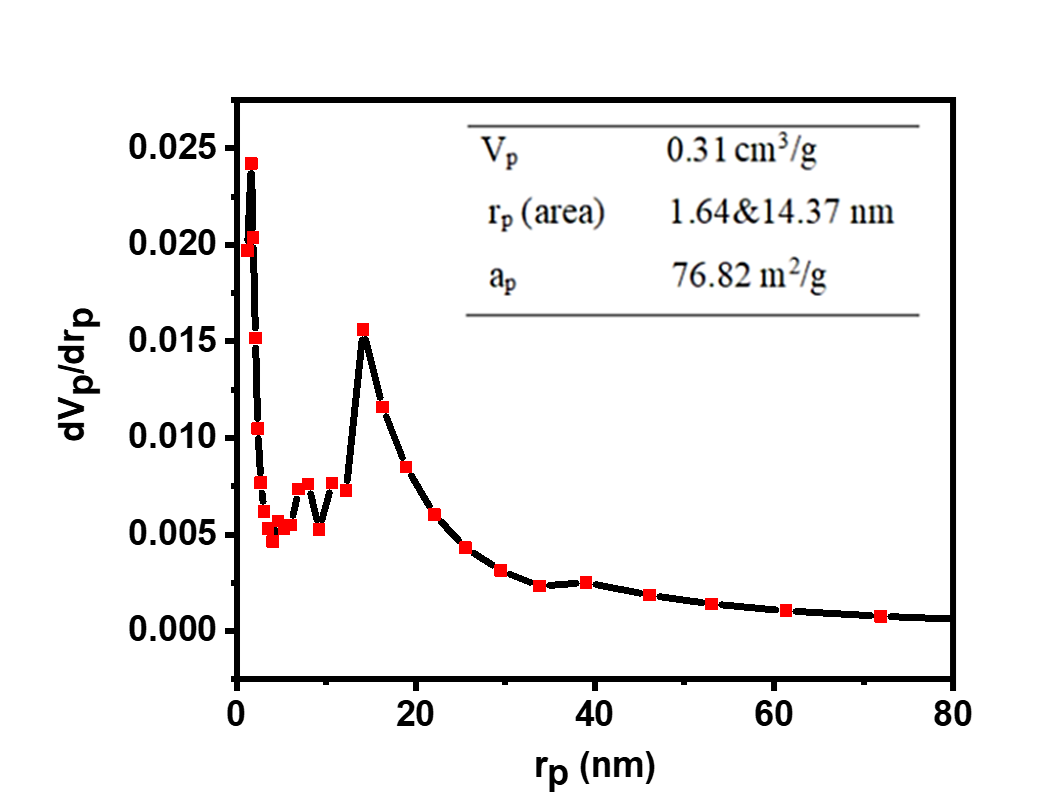


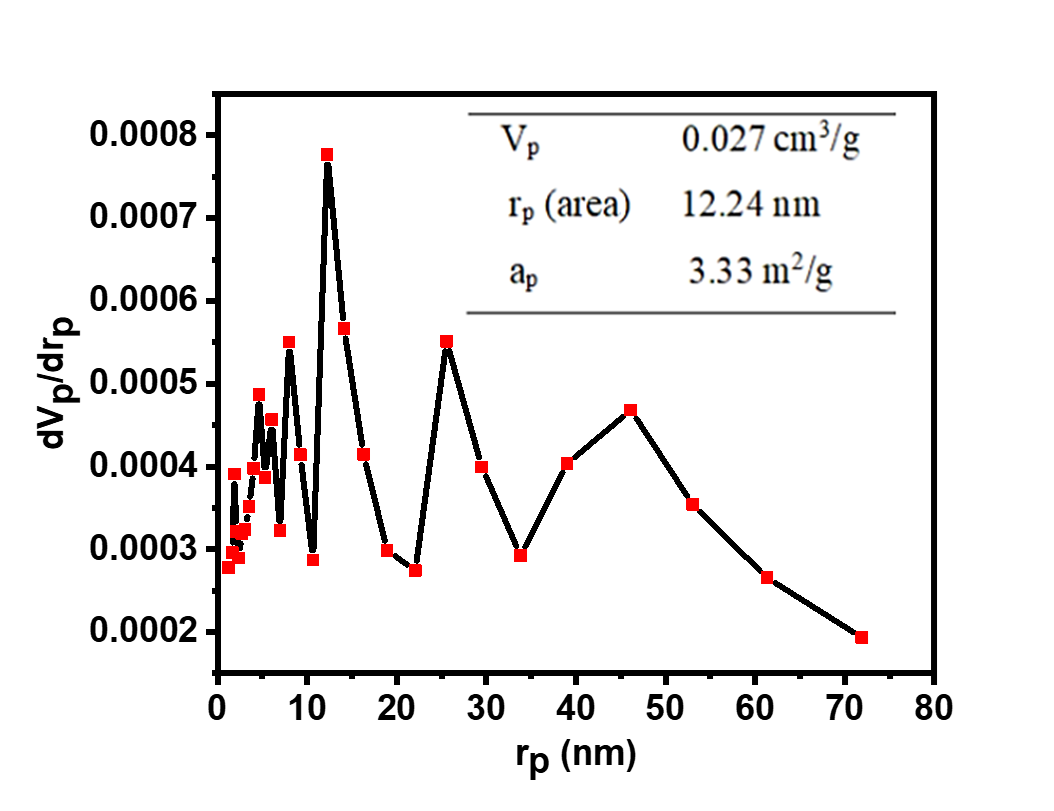


**S2.** BJH pore-size distribution of (a) SLCN and (b) bulk CN.


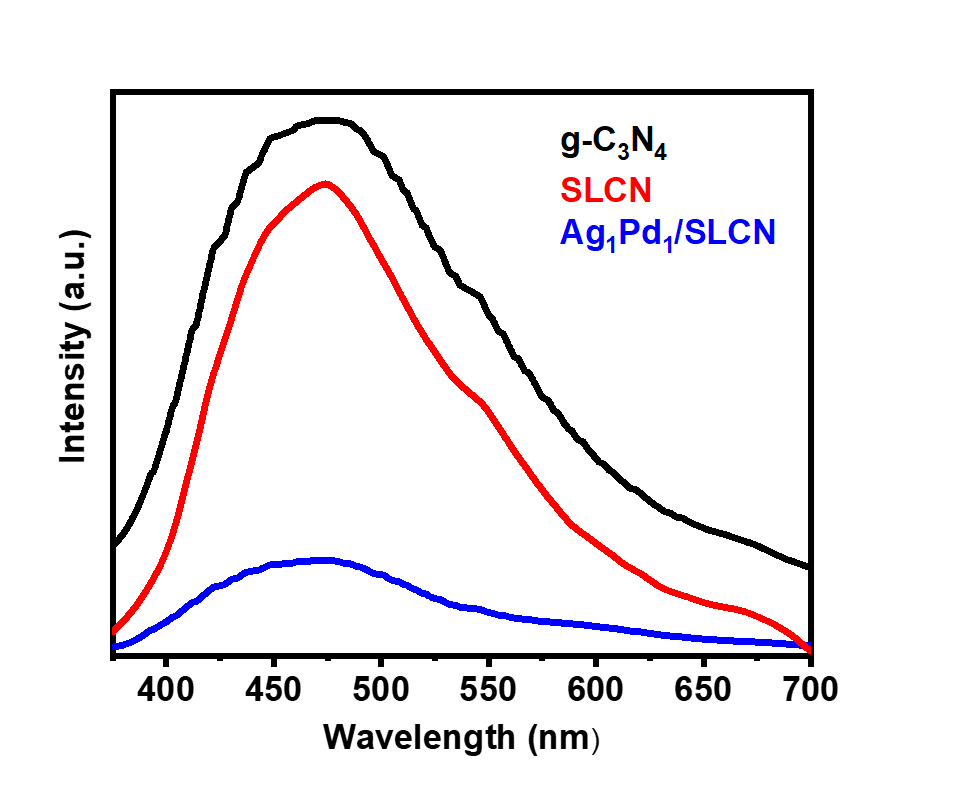


**Fig S3.** Photoluminescence (PL) spectra for SLCN and SLCN/Ag_1_Pd_1_ samples^S3^.


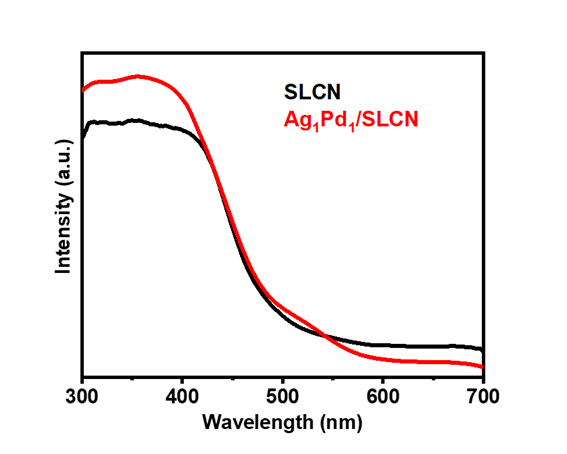


**S4.** UV-vis DRS of SLCN and Ag_1_Pd_1_/SLCN^S2,4^.

**Reference**

S1. Guo, H. *et al.* Few-layer graphitic carbon nitride nanosheet with controllable functionalization as an e ff ective metal-free activator for peroxymonosulfate photocatalytic activation : Role of the energy band bending. *Chem. Eng. J.* **401**, 126072 (2020).

S2. Yin, J.-T., Li, Z., Cai, Y., Zhang, Q.-F. & Chen, W. Ultrathin graphitic carbon nitride nanosheets with remarkable photocatalytic hydrogen production under visible LED irradiation. *Chem. Commun.* **53**, 9430–9433 (2017).

S3. Panasiuk, Y. V, Raevskaya, A. E., Stroyuk, O. L., Lytvyn, P. M. & Kuchmiy, S. Y. Preparation and optical properties of highly luminescent colloidal single-layer carbon nitride. *RSC Adv.* **5**, 46843–46849 (2015).

S4. Rong, X. *et al.* Fabrication of single-layer graphitic carbon nitride and coupled systems for the photocatalytic degradation of dyes under visible-light irradiation. *Eur. J. Inorg. Chem.* **2015**, 1359–1367 (2015).
